# Assessment of Transverse Pavement Texture Homogeneity Under Service-Stage Tire-Induced Uneven Wear Using 3D Laser Scanning

**DOI:** 10.3390/ma19132846

**Published:** 2026-07-03

**Authors:** Kunwei Zheng, Luodong Chen, Wenti Deng, Quan Lv, Man Io Leong, Difei Wu

**Affiliations:** 1 Guangdong Provincial Highway Construction Co., Ltd., Guangzhou 510600, China; 2 Key Laboratory of Road and Traffic Engineering of the Ministry of Education, Tongji University, Shanghai 201804, China

**Keywords:** pavement texture, 3D laser scanning, service stage, uneven wear, reflection intensity, weighted average grayscale, homogeneity

## Abstract

Pavement texture strongly affects skid resistance, drainage, and tire–pavement contact stability, yet its transverse evolution under wheel-track-concentrated loading remains insufficiently quantified. This study proposes a 3D-laser-scanning-based framework for evaluating the transverse homogeneity of preventive maintenance pavements during service. Ten field sections on the Jiangluo Expressway in Guangdong Province, China, covering five preventive maintenance surface systems at two service stages (six months and one year), were investigated. Reflection intensity histogram features and geometric texture parameters were screened against transverse wheel-track distribution to identify representative indicators of asphalt film peeling and aggregate wear. Weighted average grayscale was selected as the optical indicator, whereas height-distribution kurtosis was selected as the geometric indicator. A section-level homogeneity index based on normalized median absolute deviation was then used to quantify transverse dispersion. The results show that weighted average grayscale and kurtosis are the most sensitive of the tested indicators to transverse wheel-track distribution, with $R^{2}=0.973$ and R=0.9057, respectively. Wheel-track regions generally exhibited more severe optical and geometric deterioration than non-wheel-track regions, and transverse homogeneity tended to decrease from six months to one year. Within the investigated expressway sections, the framework was sensitive to different degrees of service-stage transverse wear evolution; however, broader multi-site validation is still required before threshold-based general applications can be established.

## 1. Introduction

Pavement surface texture is a key functional attribute of asphalt pavements because it directly affects skid resistance, water drainage, splash and spray control, rolling noise, and driving safety [1,2]. Macro-texture, characterized by surface wavelengths between 0.5 mm and 50 mm, primarily contributes to water drainage at high vehicle speeds, while micro-texture (wavelengths below 0.5 mm) dominates the contact adhesion between tire and pavement [1]. The combined effect of these multiscale texture features determines the overall skid resistance of the pavement surface, which is critical for ensuring driving safety, particularly under wet conditions [3]. Therefore, accurate evaluation and maintenance of pavement texture throughout its service life is of great importance for highway engineering and traffic management.

During service, however, pavement texture does not evolve uniformly across the lane width. Due to traffic channelization, wheel loads are repeatedly concentrated in limited lateral zones, causing wheel-track regions to experience more frequent compression, shear, and polishing than non-wheel-track regions [4,5]. Previous studies have shown that lateral wheel wander, typically following a normal or bimodal distribution, results in non-uniform load application across the pavement width, with approximately 60–80% of traffic loads concentrated within the wheel-track areas [4]. This repetitive concentrated loading accelerates wear in wheel-track regions, while non-wheel-track regions retain relatively higher texture levels. As a result, service-stage deterioration tends to develop as a transverse non-uniform process rather than a laterally uniform reduction in average texture, creating potential safety hazards such as hydroplaning and vehicle drift during wet weather.

This issue is especially important for preventive maintenance pavements, whose long-term effectiveness depends not only on average texture retention but also on their ability to resist localized deterioration in wheel-track regions [6,7]. Preventive maintenance treatments, including surface treatments, thin overlays, and micro-surfacing, are widely applied to restore pavement skid resistance and extend service life [8]. However, the heterogeneous wear patterns induced by traffic loading may significantly compromise their effectiveness, leading to premature functional failure in critical wheel-track zones despite adequate average performance. Recent long-term polishing studies have likewise shown that texture evolution and skid-resistance retention may diverge considerably under repeated loading, even when surfaces exhibit comparable initial functional characteristics [9]. Therefore, understanding and quantifying the transverse distribution of pavement texture deterioration under service-stage traffic loading is essential for reliable service-stage evaluation and for supporting subsequent maintenance decision-making.

Nevertheless, conventional evaluation methods remain limited in this regard. Volumetric techniques such as the sand patch test, specified in ASTM E965, provide only averaged macro-texture values (typically reported as Mean Texture Depth, MTD) without revealing lateral variations within a pavement section [10]. Although simple and widely used, these methods suffer from low efficiency, operator-dependent variability, and inability to capture local texture variations [11]. Friction tests, such as the British Pendulum Tester and Dynamic Friction Tester, measure integrated functional response without directly revealing transverse texture distribution [12]. These methods provide valuable safety-related metrics but do not distinguish between uniform and non-uniform texture conditions across the lane width. Even in studies using digital imaging or laser-based measurements, analysis often remains centered on average texture parameters rather than explicit wheel-track versus non-wheel-track differences [13,14].

Recent advances in 3D laser scanning technology provide new possibilities for refined pavement texture analysis [15,16]. Laser-based systems can acquire dense areal surface data with high horizontal and vertical resolution (typically below 0.1 mm), enabling comprehensive characterization of both macro- and micro-texture features [17]. Unlike pointwise or profile-based approaches, areal measurement mode captures complete three-dimensional surface morphology, making it particularly suitable for representing the non-uniform and spatially distributed nature of wheel-track wear. In addition to height information, the AMES 9500 scanner and similar devices can also record reflection intensity during scanning [7]. These two data domains are particularly valuable for service-stage evaluation: reflection intensity can reflect changes in binder coverage and material exposure, whereas height-field morphology captures asperity polishing, aggregate wear, and texture attenuation [18]. Recent review and sensing studies have also emphasized the growing role of automated and high-resolution texture measurement, multiscale feature extraction, and data-driven texture evaluation in pavement monitoring [19,20,21]. A service-stage assessment framework should therefore integrate both optical and geometric information rather than relying on a single average descriptor [22,23].

The mechanism of tire-induced pavement wear involves complex interaction between tire tread pattern, loading conditions, and pavement surface properties. Under repeated traffic loading, wheel contact areas experience high contact pressures and shear stresses, leading to progressive polishing of surface asperities and potential loss of texture depth [24,25]. This wear process is further complicated by environmental factors such as moisture, temperature, and contamination, which can accelerate or modify wear patterns [12]. The non-uniform nature of tire loading across the lane width, characterized by lateral wheel wander, results in differential wear rates between wheel-track and non-wheel-track regions [4]. This phenomenon creates localized zones of reduced texture and skid resistance that may not be adequately captured by average-based evaluation methods.

Despite these advances, three major gaps remain. First, most existing studies focus on average texture evolution rather than on transverse texture distribution under wheel-track-induced uneven wear [14,24]. Although several studies have quantified construction-stage aggregate-distribution homogeneity [26,27], these approaches primarily serve quality control during paving and do not address the service-stage evolution of texture non-uniformity. Second, service-stage deterioration is often interpreted from geometry alone, while the coupled role of optical response and surface morphology is not sufficiently explored [28]. Third, a practical section-level metric for quantifying the transverse homogeneity of texture features is still lacking, especially for field evaluation across heterogeneous preventive maintenance surfaces [20].

To address these gaps, this study proposes a 3D-laser-scanning-based framework for evaluating transverse pavement texture homogeneity during service. Based on field measurements from ten preventive maintenance sections on the Jiangluo Expressway in Guangdong Province, China, candidate optical and geometric features were extracted and screened against transverse wheel-track distribution to identify representative indicators of asphalt film peeling and aggregate wear. A section-level homogeneity index based on normalized median absolute deviation was then used to quantify the transverse dispersion of the selected features. The main objectives of this study are: (1) to establish a field-applicable and non-destructive framework for service-stage transverse texture evaluation; (2) to identify representative optical and geometric indicators of service-stage wear; and (3) to examine the feasibility of the proposed framework across different preventive maintenance surface systems under the investigated expressway conditions.

Compared with existing average-based texture evaluation methods, the main methodological distinction of the present study is not merely the use of 3D scanning itself, but the explicit combination of two data domains and two analysis levels. First, both reflection intensity information and 3D morphology are considered so that optical exposure and geometric reshaping can be interpreted together rather than separately. Second, the analysis is shifted from single-point or section-average descriptors to a section-level transverse homogeneity framework targeted at wheel-track-localized service-stage deterioration. In this sense, the study is intended as a feasibility-oriented complement to conventional average-texture evaluation, rather than as a replacement for friction testing, material diagnosis, or broader pavement-management decision systems.

The remainder of this paper is organized as follows. Section 2 introduces the sensing methodology and data preprocessing procedure. Section 3 presents the feature extraction strategy and homogeneity evaluation method. Section 4 describes the field experiment and test sections. Section 5 reports the results and discussion. Section 6 summarizes the main conclusions.

## 2. Methodology for 3D Pavement Sensing

### 2.1. High-Precision 3D Laser Scanning

The pavement surface data in this study were collected using the AMES Laser Texture Scanner 9500 (AMES Engineering, Ames, IA, USA), a high-resolution non-contact device developed for pavement texture measurement. The system is based on laser triangulation. During scanning, a line laser is projected onto the pavement surface, and the reflected laser line is captured by a camera positioned at a known angle relative to the laser source. Because the location of the reflected laser line shifts according to the local height variation in the surface, the pavement topography can be reconstructed through geometric triangulation. In addition to height information, the scanner also records reflection intensity, which provides useful optical information associated with binder coverage, aggregate exposure, and surface wear.

**Figure 1:**
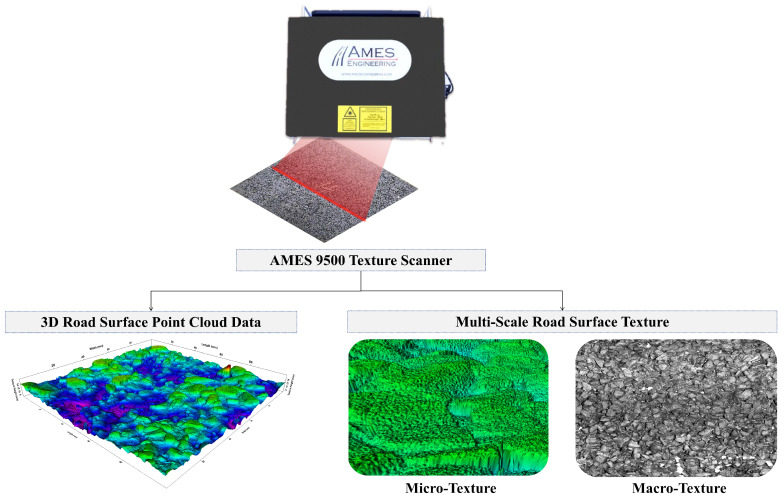
AMES 9500 scanner and measurement principle.

In the present field program, each scan covered an area of approximately 101.6mm×101.6mm, required about 90 s, and provided a vertical resolution of 0.01 mm with in-plane sampling intervals of about 0.0496 mm and 0.0415 mm in the two horizontal directions. The triangulation angle of the scanner was 18°. The AMES 9500 can therefore capture areal pavement texture data at sub-millimeter scale, which is sufficient for describing both macro-texture and fine surface morphology relevant to service-stage deterioration. Compared with conventional pointwise or profile-based approaches, the areal measurement mode of 3D laser scanning is better suited to representing the non-uniform and spatially distributed nature of wheel-track wear, and recent automated extraction and reconstruction studies have further shown the potential of laser- and LiDAR-based pavement surface acquisition for detailed areal texture analysis [29,30]. This capability is particularly important for the present study, which focuses on service-stage transverse wear and therefore requires both local surface morphology and optical-response information from multiple lateral positions within the same lane. Figure 1 shows the AMES 9500 device and the corresponding laser-triangulation measurement principle adopted in this study.

### 2.2. Transverse Sampling Strategy for Service-Stage Uneven Wear

To characterize tire-induced uneven wear across the lane width, a transverse sampling strategy was designed from the perspective of service-stage deterioration. Each test section was divided laterally into three functional regions: the left wheel-track, the middle non-wheel-track, and the right wheel-track. Ten measurement points were arranged along a single transverse section, including three points in the left wheel-track region, four points in the middle region, and three points in the right wheel-track region. The spacing between adjacent points was 20 cm.

This sampling layout was designed to reflect the concentrated nature of service-stage wear. Because traffic loading is distributed unevenly across the lane width, a single measurement point cannot adequately represent the transverse state of a pavement section. By adopting a multi-point transverse arrangement, the spatial distribution of texture features can be reconstructed and compared across different surface conditions and service times. For a given section, the extracted texture feature values can be expressed as

(1) $$X=\{X_{1},X_{2},\ldots ,X_{10}\}$$

where $X_{i}$ denotes the extracted feature value at the *i*-th transverse point. This structure supports subsequent analysis of both local differences and global section homogeneity.

**Figure 2:**
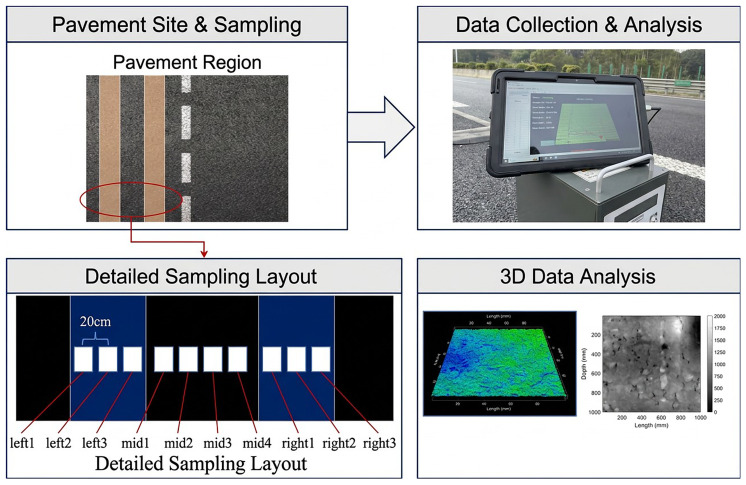
Transverse layout of ten sampling points.

Figure 2 illustrates the transverse arrangement of the ten sampling points across the lane width and the corresponding wheel-track and non-wheel-track regions considered in this study.

### 2.3. Data Preprocessing

Raw 3D scanning data may include local tilt, isolated outliers, reflection-related abnormalities, and missing values caused by occlusion or optical interference. To ensure the reliability of subsequent feature extraction, a standardized preprocessing workflow was applied.

Initially, plane correction was performed to remove background inclination caused by pavement slope or scanner placement. A reference plane was fitted to the measured surface, and the corrected height field was obtained by subtracting the fitted plane from the raw elevation data:

(2) $$z_{c}\left(x,y\right)=z_{r}\left(x,y\right)-z_{f}\left(x,y\right)$$

where $z_{r}$ is the raw height value, $z_{f}$ is the fitted reference plane, and $z_{c}$ is the corrected height.

Subsequently, the irregularly sampled point cloud was interpolated onto a regular grid to facilitate areal parameter calculation. In this study, each scanned patch was represented as a structured 100 mm × 100 mm grid using MATLAB R2025a ‘griddata’ interpolation, which preserved the dense local texture information while ensuring computational consistency for areal parameter extraction.

Furthermore, abnormal local values were removed through a two-stage denoising procedure. First, potential global outliers were screened using a skewness-adjusted outlier criterion. Second, abnormal local values and missing points were corrected by a two-dimensional median filter with a 3×3 sliding window. This procedure was necessary because service-stage pavement surfaces may exhibit isolated spikes or missing points due to strong reflection, local contamination, or geometric shadowing. The median-based local replacement suppresses these anomalies while retaining the essential morphology of the scanned surface.

Regarding reflection intensity data, grayscale normalization was additionally performed to improve comparability among different measurements. The original intensity values were transformed into grayscale values ranging from 0 to 255 as follows:

(3) $$I_{norm}\left(x,y\right)=\frac{I_{raw}\left(x,y\right)-I_{min}}{I_{max}-I_{min}}\times 255$$

where $I_{raw}\left(x,y\right)$ is the original reflection intensity at point (x,y), and $I_{max}$ and $I_{min}$ are the maximum and minimum intensity values within the scanned patch, respectively. Following normalization, threshold-based truncation was applied to reduce the influence of extreme reflective values. Based on preliminary comparison of candidate truncation thresholds between 50 and 200, a cutoff of 100 was used for the reflection intensity analysis in this study, and the subsequent histogram-based analysis focused on the 0–100 grayscale range where the vast majority of valid observations were concentrated.

**Figure 3:**
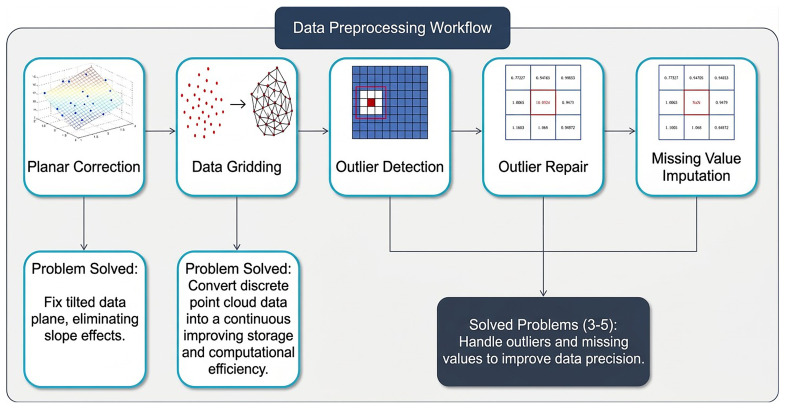
Workflow for 3D texture data preprocessing. Blue grids represent the interpolated regular data domain, and the red square highlights the target outlier or missing-value location under local-neighborhood processing.

The preprocessing workflow ensures that the subsequent optical and geometric features reflect actual pavement condition rather than measurement artifacts, providing a reliable foundation for feature extraction and homogeneity analysis. Figure 3 summarizes the overall preprocessing procedure.

**Figure 4:**
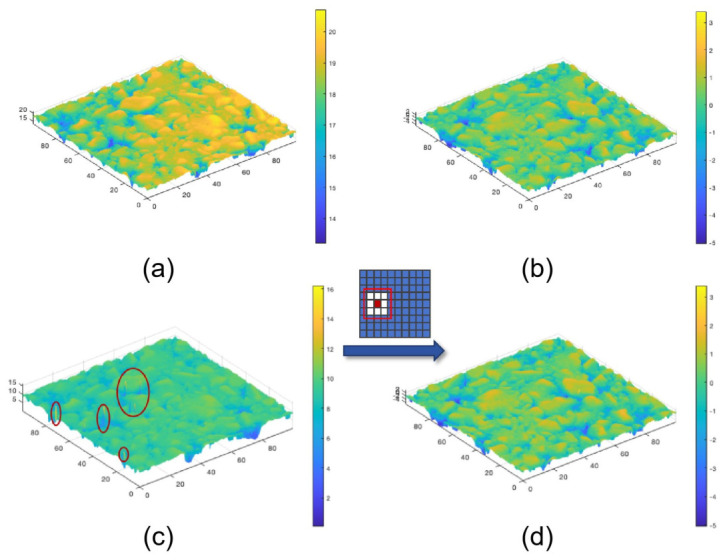
Representative preprocessing effects of 3D texture data: (**a**) tilted point-cloud data; (**b**) plane-corrected 3D point-cloud data; (**c**) 3D point-cloud data with abnormal values, where the red circles mark representative local spikes or anomalous values; and (**d**) 3D point-cloud data after abnormal-value processing. The inset grid-and-arrow schematic indicates local-neighborhood replacement during abnormal-value correction.

Figure 4 presents representative preprocessing effects and shows how the raw service-stage scans were converted into analysis-ready surface data. The comparison among raw patches, plane-corrected surfaces, local spike contamination, and median-filtered results confirms that the subsequent homogeneity analysis is based on corrected physical texture rather than on measurement artifacts.

## 3. Feature Extraction and Homogeneity Evaluation

### 3.1. Feature Extraction Framework

During the service stage, pavement texture deterioration under repeated tire action is mainly reflected in two aspects: asphalt film peeling on the surface and aggregate wear in the texture structure. Since wheel-track regions are subjected to more intensive loading than non-wheel-track regions, these deterioration processes usually exhibit obvious transverse non-uniformity. Accordingly, both optical and geometric features should be considered when characterizing the texture distribution of preventive maintenance pavements during service.

In this study, feature extraction was carried out based on the 3D laser scanning data obtained from the pavement surface. The reflection intensity information was used to characterize the optical response of the pavement surface and to reflect the surface exposure state associated with asphalt film peeling. Meanwhile, the 3D height data were used to extract geometric texture parameters, so as to characterize the morphological evolution of the pavement surface under aggregate wear.

To ensure that the extracted features are suitable for transverse distribution analysis, the candidate indicators were further screened according to their sensitivity to wheel-track distribution and their physical significance in service-stage deterioration. Based on this framework, representative optical and geometric indicators were selected for the subsequent transverse homogeneity evaluation of pavement texture.

### 3.2. Optical Features Based on Reflection Intensity Distribution

The AMES 9500 3D laser scanning system captures reflection intensity data simultaneously with pavement surface morphology acquisition. Following preprocessing and grayscale normalization, the reflection intensity information of each sampling area can be represented through a grayscale histogram. Because peeling of asphalt film during service alters the relative exposure degree of asphalt binder and aggregate, the grayscale distribution reflects optical variation in the pavement surface to a certain extent.

In this study, three histogram-based parameters were selected as candidate optical features, specifically maximum-frequency grayscale $G_{\max}$, maximum frequency $F_{\max}$, and weighted average grayscale $M_{\mathrm{weighted}}$. Among these parameters, $G_{\max}$ represents the grayscale corresponding to the histogram peak, $F_{\max}$ reflects the concentration of the peak, and $M_{\mathrm{weighted}}$ indicates the overall distribution level of the grayscale histogram.

The weighted average grayscale is calculated as follows:

(4) $$M_{\mathrm{weighted}}=\frac{\sum_{i=1}^{n}G_{i}C_{i}}{\sum_{i=1}^{n}C_{i}}$$

where $G_{i}$ is the grayscale value of the *i*-th level, $C_{i}$ is the corresponding frequency, and *n* is the number of grayscale levels.

Under repeated traffic loading, the asphalt film gradually detaches and additional aggregates become exposed on the surface, leading to a shift in the grayscale distribution. Consequently, these parameters can be utilized to characterize the optical change induced by asphalt film peeling. In the subsequent analysis, the most representative optical feature was determined according to its correlation with the transverse wheel-track distribution.

### 3.3. Geometric Features Based on 3D Surface Morphology

Beyond optical variations, pavement texture deterioration during service is also manifested as a gradual evolution of surface morphology. Under repeated tire loading, prominent asperities on the pavement surface are continuously polished and worn, which leads to changes in the height distribution of the texture. Consequently, geometric texture parameters extracted from the 3D height field can be utilized to characterize aggregate wear.

Based on corrected 3D surface data, several common texture parameters were selected as candidate geometric features, including arithmetic mean height Sa, root-mean-square height Sq, maximum peak height Sp, skewness Ssk, kurtosis Sku, developed interfacial area ratio Sdr, and mean profile depth (MPD). These parameters describe different morphological characteristics of pavement texture. Among these parameters, amplitude parameters primarily reflect the vertical scale of texture, while distribution-related parameters are more sensitive to the redistribution of surface heights caused by polishing and wear.

In particular, kurtosis Sku describes the sharpness of the surface height distribution and can better reflect the flattening effect of traffic action on texture asperities. The expression is given as

(5) $$Sku=\frac{1}{Sq^{4}}\left[\frac{1}{A}{\;\int \int \;}_{A}z^{4}\left(x,y\right)\;dx\;dy\right]$$

where z(x,y) is the height relative to the mean reference plane, *A* is the sampling area, and Sq is the root-mean-square height of the surface.

Therefore, the above geometric parameters were adopted as candidate indicators for aggregate wear characterization, and their applicability was further evaluated based on the transverse distribution characteristics of the test sections.

**Figure 5:**
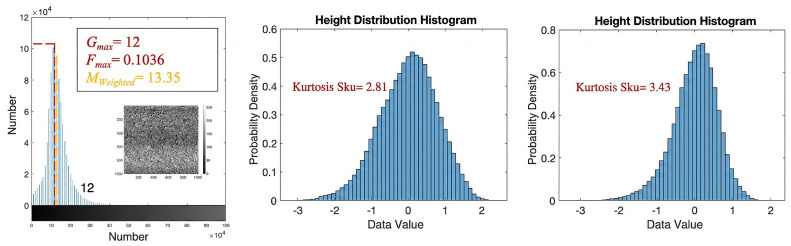
Representative interpretation of the selected optical and geometric features: (**left**), the $M_{\mathrm{weighted}}$ indicator; (**middle**), the height histogram for a non-wheel-track region; (**right**), the height histogram for a wheel-track region.

To make the physical meaning of the selected indicators more explicit, Figure 5 provides representative histogram-based examples. In the optical dimension, the weighted average grayscale reflects the global migration of the reflection intensity distribution rather than only the location or magnitude of the histogram peak. In the geometric dimension, kurtosis distinguishes relatively broad height distributions from the more concentrated distributions observed after selective wear of surface asperities. These visual interpretations further explain why $M_{\mathrm{weighted}}$ and Sku remain both statistically sensitive and physically interpretable for service-stage transverse evaluation.

### 3.4. Transverse Homogeneity Evaluation Method

Upon determination of representative optical and geometric indicators, the transverse homogeneity of pavement texture was evaluated at the section level. For each test section, ten sampling points were arranged along the transverse direction. Consequently, for a given feature, the transverse distribution within one section can be expressed as

(6) $$X=\{X_{1},X_{2},\ldots ,X_{10}\}$$

where $X_{i}$ is the feature value at the *i*-th sampling point.

To quantify the degree of transverse dispersion, the median absolute deviation (MAD) was adopted:

(7) $$\mathrm{MAD}=\mathrm{median}\left(|X_{i}-\tilde{X}|\right)$$

where n=10 and $\tilde{X}$ is the median of the feature values within the section. This median-based formulation was also the one used in the actual section-level calculations reported later for the field sections. Compared with variance-based statistics, median-based dispersion is less sensitive to isolated local disturbances and is more suitable for characterizing the transverse fluctuation of texture features under field conditions.

To eliminate the influence of magnitude differences among different features, the MAD value was further normalized:

(8) $${\mathrm{MAD}}_{n}=\frac{\mathrm{MAD}}{\tilde{X}}$$

Based on the normalized dispersion, the transverse homogeneity index was defined as

(9) $$H=-\ln({\mathrm{MAD}}_{n})$$

A larger *H* value indicates that the feature distribution is more uniform along the transverse direction, while a smaller *H* value indicates stronger transverse non-uniformity caused by wheel-track-localized deterioration.

Normalization by the section median makes the dispersion metric dimensionless and directly comparable across optical and geometric features with different units and value ranges. The negative logarithmic transformation expresses small normalized dispersions on a more readable positive scale, so that a larger *H* corresponds to higher transverse homogeneity; under this definition, a one-unit increase in *H* corresponds to an approximate *e*-fold reduction in ${\mathrm{MAD}}_{n}$. Because each section contains only ten transverse points, the median-based formulation is less sensitive than standard deviation or coefficient of variation to isolated local disturbances in field scans and is therefore used here as a comparative section-level descriptor rather than a threshold metric.

As a monotonic transformation of ${\mathrm{MAD}}_{n}$, *H* preserves the relative ordering of sections while rescaling normalized dispersion into a more interpretable index. Small numerical differences in *H* should therefore be interpreted together with the corresponding transverse distribution curves and representative 3D texture maps. Section-level results were also checked against coefficient-of-variation- and standard-deviation-based alternatives, as well as a reduced-point sensitivity calculation in which the two transition points adjacent to the wheel-track boundaries were omitted. In the present dataset, the comparative ordering remained broadly consistent, indicating that the logarithmic median-based transformation improves interpretability without materially altering the ranking pattern. The corresponding robustness results are reported in Section 5.4.

This method was applied to both selected features, enabling the transverse homogeneity of asphalt film peeling and aggregate wear to be quantitatively evaluated across different preventive maintenance surface systems and service stages.

## 4. Field Test Design and Data Acquisition

### 4.1. Test Sections and Field Scanning Procedure

Field tests were conducted on the Jiangluo Expressway in Guangdong Province, China, to investigate the transverse texture distribution of preventive maintenance pavements during the service stage. Five preventive maintenance treatments were included in the study: sand-containing fog seal, ultra-bond micro-surfacing, HET-P anti-skid surface, DTO high-ductility ultra-thin overlay, and UHPP porous wearing course.

For each treatment type, two service stages were considered, specifically six months and one year after construction. Importantly, the same ten physical sections were rescanned at both service stages, so the stage comparison is longitudinal at the section level rather than a comparison between different sections with nominally similar treatments. These sections were selected under relatively comparable operational conditions along the same expressway corridor to facilitate observation of service-stage texture evolution under heterogeneous surface conditions. Locating all sections within the same expressway corridor also reduces large between-site differences in regional climate exposure, traffic organization, and maintenance management, although it does not eliminate local variability in wheel loads, moisture condition, contamination, or construction quality. In addition, the measurements were conducted on the outer lane and under broadly comparable warm–humid environmental conditions typical of Guangdong Province, so the two campaigns are considered climatically comparable at the level required for the present longitudinal field analysis. The basic information of the investigated sections is summarized in Table 1. Since the selected treatments differ in structural form and surface characteristics, they provide diverse field cases for testing the feasibility and robustness of the proposed framework rather than merely for material ranking.

The pavement surface data were collected using the AMES 9500 high-precision 3D laser scanning system, which can simultaneously acquire surface morphology and reflection intensity information. This makes it possible to evaluate both geometric and optical characteristics of pavement texture within the same sampling area. To enhance the response to traffic-induced deterioration, the measurements were carried out mainly in the outer lane, where heavy-vehicle channelization and wheel-track effects are more evident. For each sampling area, reflection intensity data and 3D height data were recorded simultaneously, providing the basis for the subsequent analysis of asphalt film peeling and aggregate wear. The current manuscript does not include complete section-by-section traffic-volume, axle-load, or climate-monitoring records, and this absence is treated as an explicit limitation in the interpretation of treatment-specific differences.

### 4.2. Transverse Sampling Arrangement and Data Organization

To characterize the transverse non-uniformity of pavement texture, sampling points were arranged along the transverse direction of each test section. This transverse layout is shown in Figure 2. Ten sampling points were established across the lane width, covering the left wheel-track region, the middle non-wheel-track region, and the right wheel-track region. This arrangement captured lateral variation in texture features under repeated wheel loading.

At each sampling point, a local area of 100mm×100mm was scanned using the 3D laser system. The ten-point transverse sampling strategy was adopted as the basic unit for section-level texture distribution analysis. The 3-4-3 layout across the lane was chosen as a pragmatic balance between field efficiency and lateral coverage, allowing both wheel-track corridors and the middle non-wheel-track corridor to be represented within one transverse section. The wheel-track regions were defined according to the visibly worn traffic channels on the investigated outer-lane surfaces and then sampled using a fixed spacing of 20 cm. By comparing the feature values at different transverse positions, local differences between wheel-track and non-wheel-track regions were identified, while overall transverse distribution within each section was quantified.

The acquired data were organized at three levels. At the patch level, each scanned area provided one set of reflection intensity data and one set of 3D surface height data for candidate feature extraction. At the transverse-section level, feature values from the ten sampling points were combined to represent lateral distribution of texture within the section. At the section level, homogeneity indices derived from the transverse feature distributions were utilized to characterize service-stage transverse evolution and to verify the applicability of the framework under different surface conditions.

Based on this framework, subsequent analysis proceeded in three steps: extraction of candidate optical and geometric features, selection of representative indicators, and evaluation of transverse homogeneity at the section level. This data organization framework links local texture measurement with section-level distribution analysis and provides a consistent basis for analyzing service-stage texture behavior across heterogeneous preventive maintenance pavements.

## 5. Results and Discussion

### 5.1. Feature Extraction and Indicator Selection

**Figure 6:**
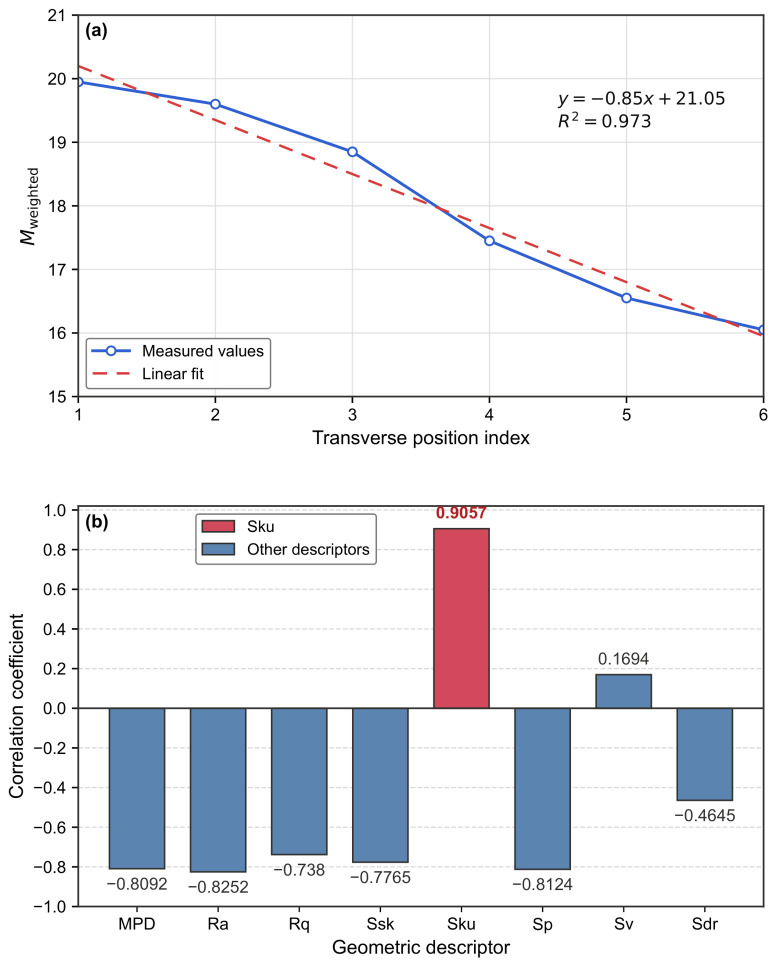
Screening evidence for the selected optical and geometric descriptors: (**a**) relationship between $M_{\mathrm{weighted}}$ and the transverse position index, where the blue solid line with circular markers shows the measured optical descriptor values and the red dashed line shows the linear fit; and (**b**) correlation coefficients of the candidate geometric descriptors with the transverse wheel-track distribution index, where the red bar highlights Sku and the blue bars represent the remaining candidate descriptors.

Indicator selection was carried out in three connected steps: visual comparison of the screening results, tabulated comparison of candidate descriptors, and final selection based on transverse sensitivity together with physical interpretability. Figure 6 provides the first layer of evidence. Panel (a) shows the fitting response of the selected optical descriptor to the transverse wheel-track distribution index, whereas panel (b) summarizes the comparative correlations of the candidate geometric descriptors with the same index. This figure therefore establishes the screening logic before the individual descriptor choices are discussed in detail.

Table 2 and Table 3 provide the second layer of evidence by listing the candidate descriptors, their physical interpretations, and the reasons for selection or exclusion. In the optical dimension, panel (a) is used to show the representative fitting result of $M_{\mathrm{weighted}}$, which was retained because it reflects the global migration of the grayscale histogram and produced the best fit to the transverse wheel-track distribution, with $R^{2}=0.973$. By contrast, $G_{\max}$ and $F_{\max}$ mainly describe local peak behavior and showed less stable transverse sensitivity, as summarized in Table 2. In the geometric dimension, panel (b) shows that several amplitude-related descriptors also responded to transverse distribution, but Sku exhibited the strongest overall association and was therefore retained as the representative geometric indicator.

#### 5.1.1. Optical Screening Identified $M_{\mathrm{weighted}}$ as the Most Responsive Descriptor

The optical screening results first indicate that service-stage reflection intensity varies systematically across the lane width. After grayscale normalization, the reflection intensity information from each sampling area was expressed as a grayscale histogram, and the histograms from different transverse positions showed clear lateral non-uniformity. In general, wheel-track regions displayed a more pronounced shift in grayscale distribution than non-wheel-track regions, indicating stronger surface exposure under repeated traffic loading.

Three histogram-based optical descriptors were then compared: maximum-frequency grayscale ($G_{\max}$), maximum frequency ($F_{\max}$), and weighted average grayscale ($M_{\mathrm{weighted}}$). Here, $G_{\max}$ represents the grayscale level at the histogram peak, $F_{\max}$ represents the concentration of that peak, and $M_{\mathrm{weighted}}$ represents the overall distribution level of the grayscale histogram. The comparison shows that $G_{\max}$ and $F_{\max}$ are more affected by local fluctuations in histogram shape, whereas $M_{\mathrm{weighted}}$ responds more consistently to the transverse loading pattern because it incorporates contributions from the full grayscale distribution. Accordingly, $M_{\mathrm{weighted}}$ provided the clearest separation between wheel-track and non-wheel-track regions and was taken to be the most suitable optical descriptor for the subsequent homogeneity analysis.

#### 5.1.2. Geometric Screening Showed That Sku Best Captures Transverse Wear Redistribution

The geometric screening results were evaluated in the same logic using corrected 3D height data. Several common texture parameters were compared, including arithmetic mean height (Sa), root-mean-square height (Sq), maximum peak height (Sp), skewness (Ssk), kurtosis (Sku), developed interfacial area ratio (Sdr), and mean profile depth (MPD). These descriptors capture different aspects of surface morphology: amplitude-related metrics such as Sa, Sq, and MPD mainly reflect the vertical scale of texture, whereas distribution-shape parameters such as Ssk and Sku are more sensitive to changes in the height distribution caused by selective wear of protruding asperities.

Among these geometric candidates, Sku showed the clearest correspondence with transverse wheel-track distribution. Its variation across lateral positions was more coherent than that of the other parameters, and the contrast between wheel-track and non-wheel-track regions was also more pronounced. This result suggests that service-stage aggregate wear should be interpreted not simply as a reduction in texture amplitude, but more fundamentally as a redistribution of surface heights under repeated wheel loading. In that sense, Sku captures the traffic-induced concentration of the height distribution more effectively than amplitude-based indicators alone.

#### 5.1.3. The Combined Screening Evidence Supports the Final Indicator Pair

Taken together, the figure-level comparison and the tabulated descriptor screening indicate that $M_{\mathrm{weighted}}$ and Sku are the most suitable optical and geometric indicators, respectively, for the present service-stage transverse evaluation framework. $M_{\mathrm{weighted}}$ was retained because it best captures the overall migration of reflection intensity distribution associated with asphalt film exposure, whereas Sku was retained because it is most sensitive to the redistribution of surface heights caused by aggregate polishing and asperity flattening. These two indicators were therefore used in the subsequent section-level homogeneity analysis.

Indicator screening was conducted as an exploratory descriptor-selection step rather than as fully independent validation. Six representative transverse positions spanning the transition from the non-wheel-track center to the wheel-track center were used to examine association with a normalized wheel-track distribution index, which serves here as a relative lateral-position surrogate rather than a directly measured traffic probability. Within this configuration, weighted average grayscale produced the strongest optical fitting relationship ($R^{2}=0.973$), and Sku showed the strongest geometric correlation (R=0.9057). The comparison therefore emphasized effect size, monotonic consistency, and physical interpretability rather than multivariable inference, and the selected indicators were subsequently applied to the ten rescanned field sections for section-level homogeneity analysis.

A leave-one-section-out check on the section-level dataset yielded the same leading descriptors: $M_{\mathrm{weighted}}$ remained the most consistently responsive optical indicator, and the geometric ranking remained led by Sku. Under the present sampling configuration, the screening results therefore support these two descriptors as the most sensitive among the tested candidates within the investigated dataset, rather than as universal ranking criteria for all pavement systems.

### 5.2. Transverse Homogeneity of Asphalt Film Peeling

Following the selection of $M_{\mathrm{weighted}}$ as the representative optical indicator, its transverse distribution within each test section was converted into a section-level homogeneity index according to the methodology described in Section 3.4. The results demonstrate that the proposed framework can identify stage-dependent transverse optical non-uniformity across sections with different surface characteristics.

In general, wheel-track regions exhibit larger deviations in $M_{\mathrm{weighted}}$ values from the section median, indicating that the optical response associated with surface exposure is more spatially concentrated in these regions. At six months, the optical homogeneity index ranged from 3.52 to 4.54, whereas at one year it decreased to 2.62–4.08, confirming an overall stage-dependent reduction in lateral uniformity. Although the magnitude of decline differed among sections, the framework consistently captured the same service-stage pattern: some sections showed pronounced reduction in homogeneity, such as 3.52 to 2.74 and 4.11 to 2.62, whereas others remained comparatively stable, such as 4.00 to 3.97 and 3.61–4.08 at one year. These heterogeneous responses demonstrate that the proposed optical indicator remains interpretable across multiple preventive maintenance surface systems rather than being limited to a single pavement type.

A comparative analysis between the two service stages further indicates that the transverse homogeneity of asphalt film peeling generally decreases from six months to one year. This suggests that optical deterioration of the pavement surface becomes increasingly laterally non-uniform with the accumulation of traffic loading. In other words, asphalt film peeling during the service stage does not develop as a uniform process across the lane width but rather exhibits an increasingly localized tendency in wheel-track regions. At the same time, reflection intensity should be interpreted as an indirect optical proxy rather than a direct one-to-one measurement of asphalt film peeling, because it may also be influenced by surface contamination, moisture condition, material color, and local mineralogical differences. The optical results should therefore be interpreted as texture-related evidence of service-stage surface exposure rather than as direct proof of binder loss.

**Figure 7:**
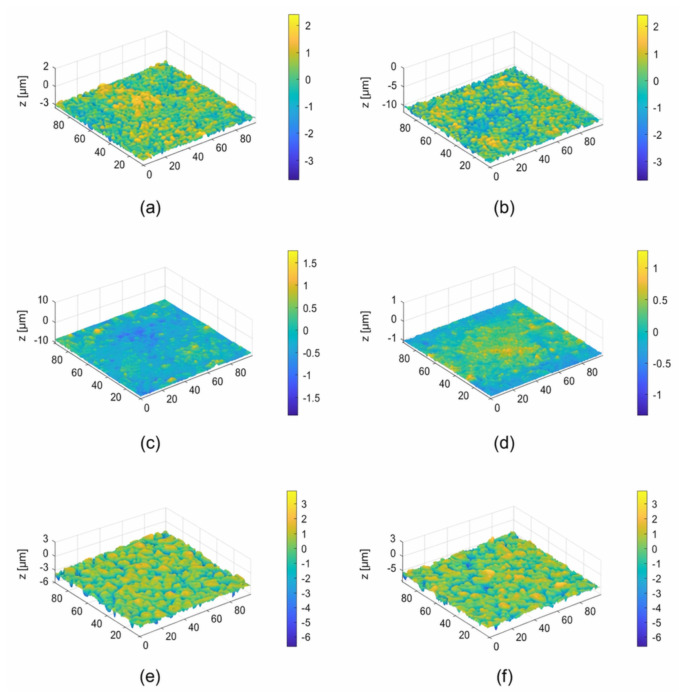
Representative 3D texture evolution of preventive maintenance surfaces: (**a**,**b**), sand-containing fog seal at six months and one year after treatment, respectively; (**c**,**d**), ultra-bond micro-surfacing at six months and one year after treatment, respectively; and (**e**,**f**), HET-P anti-skid surface at six months and one year after treatment, respectively.

To complement the section-level homogeneity index, Figure 7 presents representative 3D texture maps for three preventive maintenance surface systems. The side-by-side comparison of six-month and one-year surfaces provides direct visual evidence of how wheel-track-localized wear evolves on the pavement surface and how the degree of texture retention differs across treatment types.

**Figure 8:**
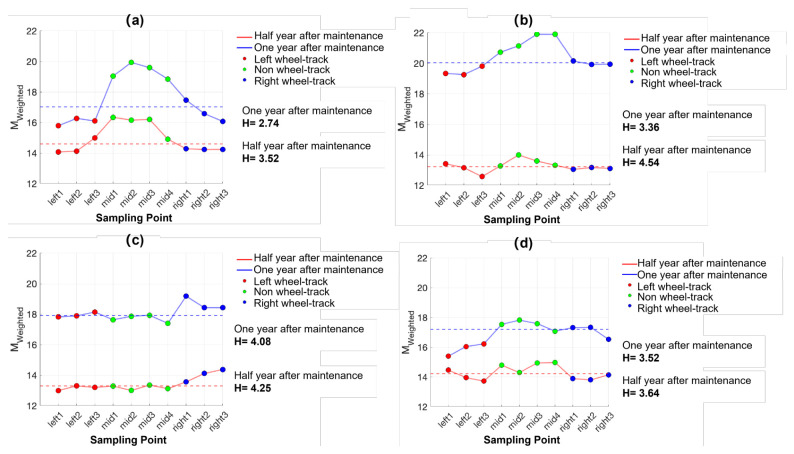
Representative transverse distributions of $M_{\mathrm{weighted}}$ and the corresponding optical homogeneity indices for selected preventive maintenance treatments: (**a**) sand-containing fog seal; (**b**) ultra-bond micro-surfacing; (**c**) UHPP porous wearing course; and (**d**) DTO ultra-thin overlay.

Figure 8 further presents representative transverse distribution curves and retains the lateral-position information that is compressed when the analysis is reduced to one section-level *H* value. The selected examples are organized by treatment type so that the characteristic transverse response of each representative surface system can be compared directly. Specifically, panels (a)–(d) correspond to sand-containing fog seal, ultra-bond micro-surfacing, UHPP porous wearing course, and DTO ultra-thin overlay, respectively. By plotting the ten transverse sampling points for these representative cases, the characteristic wheel-track-localized patterns can be directly observed and linked to the corresponding homogeneity indices.

Table 4 provides a compact numerical summary of the robustness checks for the section-level homogeneity index *H*, complementing the subsequent graphical comparison in Figure 10.

**Figure 9:**
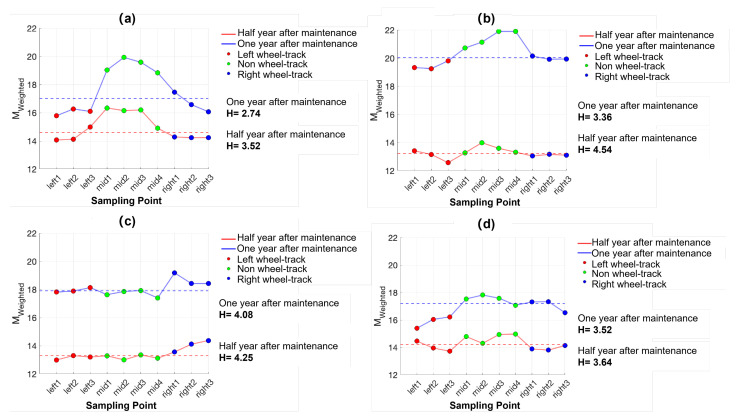
Section-level transverse optical homogeneity indices derived from $M_{\mathrm{weighted}}$ at six months and one year.

Figure 9 compares the section-level optical homogeneity indices at the two service stages. The figure presents the section-level *H* values derived from $M_{\mathrm{weighted}}$ and makes it possible to compare framework-detected differences in wheel-track-localized asphalt film peeling under multiple surface conditions.

Because each section-level *H* value is derived from only ten transverse points, local sensitivity and uncertainty were further examined. Bootstrap resampling yielded mean half-widths of about 1.29 for the optical index and 1.28 for the geometric index, whereas leave-one-point-out calculations gave markedly smaller average standard deviations of 0.18 and 0.21, respectively. Absolute *H* values should therefore not be interpreted as threshold-like constants, whereas comparative section ordering is more stable. The ordering also remained strongly aligned with alternative dispersion summaries based on coefficient of variation and standard deviation, indicating that the logarithmic median-based transformation mainly improves interpretability rather than changing the ordering structure.

**Figure 10:**
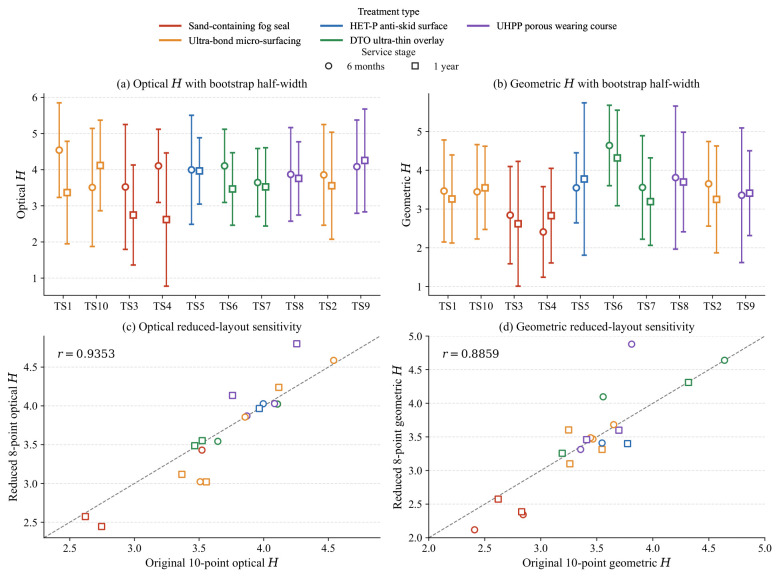
Robustness checks for the section-level homogeneity index *H*. Panels (**a**,**b**) show the bootstrap half-widths of the optical and geometric *H* values for the investigated sections at the two service stages. Panels (**c**,**d**) compare the original 10-point results with the reduced 8-point sensitivity results obtained after omitting the two transition points adjacent to the wheel-track boundaries.

Figure 10 shows the same pattern visually. The bootstrap panels indicate non-negligible uncertainty ranges when only ten transverse points are available, whereas the reduced-layout comparison preserves strong correspondence for both optical and geometric results. The proposed index is therefore better suited to comparative section-level discrimination than to threshold-style interpretation of a single absolute *H* value.

Stage-dependent change also remained heterogeneous across sections. A paired Wilcoxon signed-rank comparison of the ten rescanned sections yielded a downward tendency in the optical homogeneity index from six months to one year (median paired change −0.211, rank-biserial effect size −0.636, p=0.084), whereas the corresponding geometric comparison showed a weaker downward tendency (median paired change −0.160, rank-biserial effect size −0.309, p=0.4316). These results are consistent with increasing wheel-track-localized non-uniformity at the descriptive level, but they do not imply a uniform statistical rule across all investigated treatments.

### 5.3. Transverse Homogeneity of Aggregate Wear

After Sku was selected as the representative geometric indicator, its transverse distribution was further converted into a section-level homogeneity index in the same manner as the optical feature. The results indicate that the homogeneity of aggregate wear also decreases in the wheel-track direction, but the geometric response provides a more direct description of the redistribution of surface heights caused by repeated tire polishing. Compared with the middle non-wheel-track region, the wheel-track zones generally exhibit higher Sku values, indicating that the height distribution becomes sharper and more concentrated as prominent asperities are progressively worn down.

The correlation analysis conducted during indicator screening provides important support for this interpretation. Among the candidate geometric parameters, Sku showed the strongest relationship with transverse wheel-track distribution, with a correlation coefficient of R=0.9057. Although MPD, Ra, and Sp also exhibited relatively strong correlations, with absolute correlation coefficients above 0.8, these amplitude-oriented indicators mainly reflect the loss of texture magnitude. By contrast, Sku better captures the reshaping of the height distribution, which is the more characteristic manifestation of service-stage aggregate wear under repeated traffic action.

At the section level, better optical homogeneity generally coincided with more stable geometric homogeneity, suggesting that asphalt film peeling and aggregate wear are related rather than independent processes. The geometric homogeneity index ranged from 2.48 to 4.64 at six months and from 2.23 to 4.32 at one year, with some sections showing clear decline and others remaining comparatively stable. This variation indicates that the Sku-based homogeneity analysis can distinguish different degrees of wheel-track-localized geometric deterioration across the investigated preventive maintenance surfaces. The observed trends are consistent with a linked progression in which optically exposed wheel-track zones become more susceptible to subsequent polishing and flattening, although the present dataset remains descriptive and does not establish a universal transition threshold for such coupling [31].

The service-stage comparison further suggests that geometric homogeneity generally decreases from six months to one year, which is consistent with the accumulated effect of channelized traffic. This trend implies that the geometric non-uniformity of the pavement surface is not formed instantaneously after treatment construction, but develops progressively as the wheel-track regions experience repeated contact stress and shear action. Therefore, the use of Sku-based homogeneity analysis provides a useful supplement to conventional depth-based indicators when evaluating the long-term anti-wear capacity of preventive maintenance pavements, while still requiring cautious interpretation when section-to-section variability is large.

**Figure 11:**
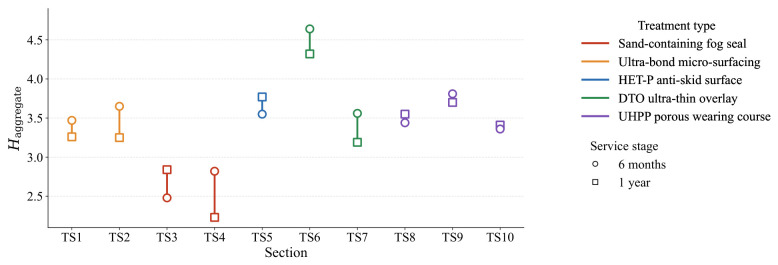
Section-level transverse geometric homogeneity indices derived from Sku at six months and one year.

Figure 11 compares the corresponding geometric homogeneity indices. The figure summarizes the section-level *H* values calculated from Sku and highlights the framework-detected differences in transverse aggregate wear evolution across the investigated surface systems.

### 5.4. Discussion on Framework Validation Across Multiple Surface Types

Table 5 summarizes the treatment-specific transverse response patterns observed across the investigated surface systems and provides a compact comparison of how the five preventive maintenance treatments evolved between the two service stages.

The results confirm that the proposed framework captures two complementary manifestations of service-stage deterioration. In the optical dimension, the weighted average grayscale $M_{\mathrm{weighted}}$ exhibited the clearest relationship with wheel-track distribution, with a coefficient of determination of $R^{2}=0.973$. This result indicates that optical deterioration is mainly expressed as a global migration of the reflection intensity histogram rather than as an isolated change in the histogram peak. In practical terms, the traffic-induced loss of asphalt film affects the overall grayscale composition of the scanned surface, and this global shift is better represented by $M_{\mathrm{weighted}}$ than by peak-based descriptors such as $G_{\max}$ or $F_{\max}$. As summarized in Figure 9 and Table 5, the optical homogeneity index decreased from 3.52 to 4.54 at six months to 2.62–4.08 at one year, indicating that lateral non-uniformity became more evident with increasing service time.

In the geometric dimension, the superiority of Sku over conventional amplitude parameters indicates that repeated tire action should be interpreted as a redistribution process of the surface height field rather than merely a reduction in texture depth. This distinction is important because two surfaces may show comparable average depth while still exhibiting very different transverse wear patterns. Similar observations have also been reported in multiscale 3D texture analyses used for pavement-friction prediction [32]. The present results therefore suggest that service-stage texture evaluation should move beyond average texture retention and pay more attention to whether the transverse profile remains laterally balanced under wheel-track loading. Figure 11 further shows that the geometric homogeneity index spanned 2.48–4.64 at six months and 2.23–4.32 at one year, again confirming the progressive concentration of wear in wheel-track regions.

Clear differences in transverse response were observed among the investigated sections. Sand-containing fog seal showed the strongest stage-dependent wheel-track localization, ultra-bond micro-surfacing and DTO ultra-thin overlay showed moderate decline, and UHPP porous wearing course remained comparatively stable. The framework therefore distinguishes different degrees of service-stage transverse evolution across the investigated preventive maintenance surfaces, while not supporting a treatment ranking independent of local section conditions.

Optical and geometric trends were broadly consistent. Sections with stronger optical non-uniformity generally showed stronger geometric deterioration, supporting the interpretation that asphalt film exposure and aggregate polishing are related service-stage processes. This agreement indicates that combined optical and geometric indicators provide a more robust basis for identifying transverse wear evolution than either dimension alone [9,32].

The selected indicators should be interpreted as mixed-scale descriptors extracted from the scanned patch rather than as strictly separated macro-texture or micro-texture parameters, because no wavelength-band decomposition was applied before feature extraction. The framework is likewise best understood as a surface-response evaluation approach rather than a complete material-diagnosis tool: reflection intensity change and height-distribution reshaping capture informative manifestations of service-stage wear, but they do not independently resolve binder aging, aggregate mineralogy, adhesion loss, moisture damage, or sub-surface heterogeneity.

The field validation covered ten sections from five treatment types and two service stages on a single expressway in Guangdong Province, China. This is sufficient to demonstrate methodological feasibility, but still limited for establishing broader deterioration thresholds or climate-independent treatment rankings. The proposed indices were not directly coupled to friction, hydroplaning, or accident-risk metrics, and section-level repeatability and preprocessing sensitivity were not systematically quantified. Although bootstrap and leave-one-point-out checks improve confidence in comparative section ordering, full repeatability testing, environmental normalization, and direct functional validation remain necessary in future work.

## 6. Conclusions

This study proposed a 3D-laser-scanning-based framework for evaluating the transverse homogeneity of preventive maintenance pavements during the service stage by integrating reflection intensity information and 3D surface morphology to characterize asphalt film peeling and aggregate wear under wheel-track-induced uneven loading. Based on repeated field measurements from ten physical sections of the Jiangluo Expressway in Guangdong Province, China, weighted average grayscale $M_{\mathrm{weighted}}$ and kurtosis Sku were identified as the most sensitive of the tested optical and geometric indicators to transverse wheel-track distribution, with $R^{2}=0.973$ and R=0.9057, respectively. Wheel-track regions generally exhibited more severe optical and geometric degradation than non-wheel-track regions, and the homogeneity of both dimensions tended to decrease from six months to one year. The section-level homogeneity index based on normalized median absolute deviation provided an interpretable measure of transverse texture dispersion in the investigated sections. Application to five preventive maintenance surface systems further showed that the framework can distinguish different degrees of service-stage transverse evolution beyond conventional average-based texture indicators. The present results remain a field-based feasibility demonstration rather than a universal treatment-ranking framework, because the dataset is limited to ten sections on a single expressway and the proposed indices have not yet been directly linked to friction measurements, repeatability statistics, or safety-performance indicators. Future work should therefore expand the field dataset and establish direct relationships between transverse texture homogeneity, friction evolution, and service performance.

## Figures and Tables

**Table 1:** Basic information of the ten physical field sections used for service-stage texture homogeneity evaluation. Each section was rescanned at both service stages.

Section ID	Treatment	Chainage	Service-Stage Observations
TS1	Ultra-bond micro-surfacing	K189+800	Same section rescanned at both stages
TS2	Ultra-bond micro-surfacing	K215+300	Same section rescanned at both stages
TS3	Sand-containing fog seal	K239+670	Same section rescanned at both stages
TS4	Sand-containing fog seal	K289+000	Same section rescanned at both stages
TS5	HET-P anti-skid surface	K213+160	Same section rescanned at both stages
TS6	DTO high-ductility ultra-thin overlay	K271+000	Same section rescanned at both stages
TS7	DTO high-ductility ultra-thin overlay	K291+100	Same section rescanned at both stages
TS8	UHPP porous wearing course	K191+460	Same section rescanned at both stages
TS9	UHPP porous wearing course	K192+500	Same section rescanned at both stages
TS10	UHPP porous wearing course	K230+915	Same section rescanned at both stages

**Table 2:** Candidate optical descriptors considered during the indicator-screening stage.

Descriptor	Interpretation	Screening Result	Decision
$G_{\max}$	Peak grayscale location	Weaker transverse consistency than $M_{\mathrm{weighted}}$	Excluded
$F_{\max}$	Peak concentration of histogram	Sensitive to local histogram fluctuation	Excluded
$M_{\mathrm{weighted}}$	Overall grayscale distribution level	Best fit to wheel-track distribution, $R^{2}=0.973$; captures whole-histogram migration	Selected

**Table 3:** Candidate geometric descriptors considered during the indicator-screening stage.

Descriptor	Interpretation	Screening Result	Decision
MPD	Mean profile depth	Strong but secondary association with transverse distribution, R=−0.8092	Excluded
Ra	Arithmetic roughness amplitude	Strong but secondary association with transverse distribution, R=−0.8252	Excluded
Rq	Root-mean-square roughness amplitude	Moderate-to-strong association with transverse distribution, R=−0.7380	Excluded
Ssk	Height-distribution skewness	Moderate association with transverse distribution, R=−0.7765	Excluded
Sku	Height-distribution kurtosis	Strongest geometric association, R=0.9057; best reflects height-field redistribution	Selected
Sp	Maximum peak height	Strong but secondary association with transverse distribution, R=−0.8124	Excluded
Sv	Maximum pit depth	Weak association with transverse distribution, R=0.1694	Excluded
Sdr	Developed interfacial area ratio	Limited association with transverse distribution, R=−0.4645	Excluded

**Table 4:** Compact robustness summary of the section-level homogeneity index *H* for the investigated dataset. Bootstrap intervals were calculated from the ten transverse points within each section, and leave-one-point-out statistics were used to assess local ranking stability.

Indicator	Mean Bootstrap Half-Width of *H*	Mean Leave-One-Point-Out s.d. of *H*	Additional Robustness Observations
$M_{\mathrm{weighted}}$	1.29	0.18	The correlation between *H* and the inverse normalized dispersion remained strong, and the section ordering under the original 10-point layout remained highly consistent with a reduced 8-point sensitivity calculation (r=0.9353).
Sku	1.28	0.21	The section-level geometric ranking was likewise broadly preserved under leave-one-point-out analysis, and the reduced 8-point sensitivity calculation still remained strongly correlated with the original 10-point ordering (r=0.8859).

**Table 5:** Section-level treatment-response summary for the investigated expressway sections. The statements describe observed patterns rather than general material rankings.

Surface System	Observed Treatment-Specific Response
Sand-containing fog seal	Among the investigated sections, this treatment shows the clearest decline in both optical and geometric homogeneity. A plausible explanation is the relatively thin surface film and weaker retention of surface particles under repeated wheel-track loading, although dedicated material-level verification is still needed.
Ultra-bond micro-surfacing	The investigated sections show optical and geometric decline from six months to one year, but the reduction remains moderate rather than abrupt. This response suggests measurable wheel-track localization while retaining greater short-term stability than sand-containing fog seal.
HET-P anti-skid surface	The investigated section shows only limited change between the two service stages and remains comparatively stable in both dimensions over the present observation window.
DTO ultra-thin overlay	The investigated sections still show stage-dependent decline, but the geometric response remains comparatively stable relative to the more deterioration-prone sections in this dataset.
UHPP porous wearing course	The investigated sections vary only slightly between stages in both optical and geometric homogeneity, indicating comparatively balanced transverse behavior under the tested expressway conditions.

## Data Availability

The data presented in this study are available on reasonable request from the corresponding author. The data are not publicly available because they were obtained from field measurements on an operating expressway and are subject to project-related access restrictions.
